# Progressive Politics and Humanistic Psychology in the Trump/Coronavirus Era

**DOI:** 10.1177/0022167820934226

**Published:** 2020-11

**Authors:** Elliot Benjamin

**Affiliations:** 1 Capella University, Minneapolis, MN, USA

**Keywords:** humanistic psychology, coronavirus pandemic, self-care, progressive politics, Resisting Trump movement, Trump/Coronavirus era

## Abstract

In this article, the author discusses the relationship of progressive politics to humanistic psychology in the Trump/Coronavirus era. The harsh realities of personal fears and severe challenges to our mental health evoked by both the United States presidency of Donald Trump and the coronavirus pandemic are described initially. Then, a number of self-care practices that are consistent with the basic values of humanistic psychology and that we can undertake to help us meet these harsh realities are illustrated. Next, the author describes his own personal engagement and self-care in the world of progressive politics and humanistic psychology in the context of the Resisting Trump movement. The article concludes with the author suggesting that perhaps it may be worthwhile for politically like-minded others to also consider finding ways of merging their progressive politics with humanistic psychology in order to enhance their self-care through these turbulent times in the Trump/Coronavirus era.

## Introduction

In the present article, I want to focus on the merging of progressive politics and humanistic psychology during the tremendously stressful and dangerous times in which we are currently living, which I refer to as the Trump/Coronavirus era. The merging that I describe in this article is based on my own relevant experiences, primarily in the United States, but they may very well have applications to various countries in other parts of the world. I have published a number of articles and given a number of talks related to the merging of progressive politics and humanistic psychology in the context of the Resisting Trump movement (Benjamin, 2018a, 2018b, 2019).^1^ In the concluding section of one of my articles (Benjamin, 2019), I summarized my view that many of the statements and policies of United States President Donald Trump are completely antithetical to the basic premises of humanistic psychology that involve engaging in genuine and empathic relationships with people:

Under the current dangerous leadership and rhetoric of US President Donald Trump, the destructive effects on the mental health of much of our country’s population are becoming increasingly apparent. Furthermore, President Trump’s various political activities, especially inclusive of his dangerous rhetoric, may very well have led to significant increases in violence, and they run completely counter to the basic values of Humanistic Psychology that promote genuine, empathic relationships between human beings. (pp. 7-8)

My above summary describing the detrimental effects of Trump’s leadership and rhetoric on mental health, as well as its apparent stimulation of significant increases in violence (Benjamin, 2019), is supported by a number of quantitative statistical studies as conveyed by various authors: (Fein et al., 2018; Finkelstein et al., 2018; Folley, 2019; Haverluck, 2018; Macias, 2019; Panning, 2019).

However, the concerns that myself and others have previously written about in regard to the “destructive effects on the mental health of much our country’s population” under the “dangerous leadership and rhetoric of US President Donald Trump” (Benjamin, 2019, pp. 7-8) are currently greatly increased due to the current coronavirus pandemic crisis.^2^ Nevertheless, I believe that the merging of progressive politics and humanistic psychology in the context of the Resisting Trump movement, now applied in our current Trump/Coronavirus era, may therapeutically enhance our mental health while literally saving the United States and the world from the point of disaster with no escape (Benjamin, 2020a, 2020b, 2020c, 2020d). To support my contention about the value of this merging of progressive politics and humanistic psychology, I will make use of my own relevant experiences in the Resisting Trump movement with a current focus on the coronavirus pandemic crisis.

## Detrimental Effects on Mental Health in the Trump/Coronavirus Era

There have been a number of disturbing reports about the significant detrimental effects on the mental health of a large segment of the U.S. population, directly related to our present political climate under what I have referred to as the “dangerous leadership and rhetoric” of President Donald Trump (Benjamin, 2019). One of these disturbing reports conveyed the following:

Trump’s behaviors during the campaign (e.g. misogyny, and ethnic bigotry, prejudice, and discrimination), and his subsequent success have ignited and perpetuated feelings and beliefs characterized by fear of and hostility toward particular groups, and major shifts in beliefs and perspectives that reveal potentially significant challenges people could face as a result of the disruption of a particular worldview. . . . Specifically, the demonstrated threats to human rights and civil liberties have raised disturbing challenges for many American citizens and undocumented immigrants in the form of deep concern at being led and governed by an individual who does not promote public safety, equality, and human dignity in language and behavior. (Krippner & Pitchford, 2018, pp. 175-176)

The prevalent anxiety that many Americans have experienced since the 2016 election of Trump has been referred to as “Trump Anxiety Disorder” and described as follows:

Symptoms were specific to the election of Trump and the resultant unpredictable sociopolitical climate. . . . Though not an official diagnosis, the symptoms include feeling a loss of control and helplessness, and fretting about what’s happening in the country and spending excessive time on social media. . . . Trump is driving the nation into a mental crisis. “Two-thirds of Americans say they are stressed about the future of our nation—including a majority of Democrats and Republicans,” an APA press release issued last year revealed. “More than half of Americans (57 percent) say the current political climate is a very or somewhat significant source of stress, and nearly half (49 percent) say the same about the outcome of the election.” . . . “We’re surrounded by conversations, news and social media that constantly remind us of the issues that are stressing us the most.” (Haverluck, 2018, pp. 3-5)

Furthermore, as I have previously described, the precarious mental health of President Trump himself may very well be a significant factor related to these detrimental mental health effects:

A significant factor in this detrimental mental health phenomenon in the United States is directly related to the questionable mental health of President Trump himself, as strikingly described in terms like malignant, pathological, hedonistic, and narcissistic by a number of well-respected psychiatrists and psychologists in the book *The Dangerous Case of Donald Trump* (Lee, 2019). (Benjamin, 2019, p. 4)

However, these disturbing reports pale by comparison with what the whole world is now facing with the current coronavirus pandemic crisis, and the United States is doubly challenged by what I have referred to as the “Deadly Duo”: Trump and the coronavirus (Benjamin, 2020a, 2020b, 2020c). The deadly worldwide consequences of the coronavirus have been described excessively,^2^ and there have also been a number of descriptions of the detrimental effects on mental health in the United States from this crisis (Benjamin, 2020d; Graham, 2020; Holcombe, 2020; Hunter, 2020; Kaur, 2020). Some of these descriptions are as follows:

Along with these feelings of disorientation, it may seem like it’s getting harder to concentrate and taking longer to complete tasks, as if our brains are just working more slowly. . . . “It’s a perfect storm between changes in environment, loss of social anchors and increases in cognitive stress. . . . And then on top of that, most of us are not getting the quality sleep that we used to” . . . “Often if you’re feeling stressed or you’re feeling anxious, those thoughts and feelings can show up and either make it more difficult to fall asleep or more difficult to stay asleep.” . . . That sleeplessness, in turn, can further cognitive impairment, attention and concentration issues and short-term memory loss. . . . “We’ve lost all of the routine of a typical week, and that means having weekends as a boundary or as a separation or something to look forward to. . . . Now the weekend is the same as a weekday. Because work is home and home is work for many people, some may find themselves working longer hours or into the weekends. Gone are the happy hours, concerts or sporting events that once separated weekdays from weekends, causing the days to just drag on. . . . For those who still need to go into work, routines may look a lot different. And there’s the added mental strain of remembering to socially distance, wear masks and avoid touching surfaces. All of that can contribute to a sense of disorientation.” . . . We’re multitasking a lot more. Many people are finding themselves balancing multiple responsibilities, such as homeschooling children or caring for an elderly family member—all while holding down a full-time job or coping with the stress of a layoff. . . . “Our working memory is a limited resource. . . . We can easily tax it by trying to engage it in too many activities at once or trying to multitask in our mind.” The pandemic is becoming a source of chronic stress given that it’s been going on for weeks, or even months for some people. . . . High stress levels impair our concentration and attention, and can affect short-term memory. (Kaur, 2020, pp. 1-3)

“We’ve lost that sense of certainty, that sense of safety, that sense of predictability and so it stands to reason that all of that leaves us feeling dislocated and unsure about what’s going to happen next.” . . . People all over the world are grieving the sudden loss of loved ones, and the intensity of those losses is clear. But grief can come from the loss of anything we’re attached to deeply: the loss of economic stability, the loss of our ability to move around freely, the ability to participate in life’s milestones in person. (Hunter, 2020, p. 1)

Across the country, seniors’ lives are being upended as continuing care retirement communities take aggressive steps to protect residents from Covid-19, the illness caused by the novel coronavirus. . . . Without regular contact with other people, older adults can become lonely or depressed. A change in someone’s health status that might have been noticed if they didn’t show up for dinner can now go unobserved. Without stimulation, motivation and cognition can decline. (Graham, 2020, pp. 1, 4)

It could put strain on families, send children home to abusive situations, make those living alone feel isolated and threaten people’s sense of purpose by keeping them from work. . . . And those experiencing financial insecurity in the midst of the pandemic have an added stress that is difficult to resolve. . . . The experience of staying home together through a pandemic can be considered a collective trauma. . . . People in quarantine show signs of confusion, depression and anger. (Holcombe, 2020, p. 2)

It is well-known that cognitive stimulation is significantly related to preventing cognitive decline (Eckroth-Bucher, 2009; Krell-Roesch et al., 2019), and the related detrimental consequences to mental health from the coronavirus are likely spreading throughout the world. In particular, in China, there are studies that have reported detrimental mental health consequences that include anxiety, depression, sleep-related symptoms, and fears of being quarantined and isolated from the world (Huang & Zhao, 2020; Wilder-Smith & Freedman, 2020). However, as described in the next section, there are also a number of self-care recommendations from health care professionals that may offset at least some of the detrimental mental health consequences from the coronavirus, and some of these recommendations are consistent with the basic premises of humanistic psychology (C. R. Rogers, 1961).

## Self-Care, Humanistic Psychology, and the Coronavirus Crisis

Mental health professionals have recommended a number of self-care practices to offset the detrimental mental health consequences of the coronavirus. These recommended self-care practices include developing self-awareness, self-acceptance, and self-compassion, practicing mindfulness, meditation, and/or yoga, connecting with others “virtually,” engaging in constructive and cathartic self-expression, limiting the frequency of getting news updates, limiting social media time, obtaining professional “virtual” mental health assistance, getting adequate sleep, keeping up with proper nutrition, getting outside as much as possible, engaging in regular physical activity, spending enough alone-time if living with someone, focusing on the joys in the little things in life, maintaining a sense of daily structure as much as feasible, and taking frequent breaks (Graham, 2020; Holcombe, 2020; Hunter, 2020; Kaur, 2020; K. Rogers, 2020; Villano, 2020; Willingham, 2020). A number of these self-care practices have been well-established as beneficial practices to mental health in a variety of circumstances (Brenner, 2014; Kabat-Zinn, 2005; C. R. Rogers, 1961; Schneider et al., 2015). The following illustrates some of these recommendations that are consistent with the basic premises of humanistic psychology that involve self-awareness, self-expression, creativity, authenticity, and empathy:

[Senior] communities have responded by having staff check in regularly with vulnerable residents, offering to arrange video visits with family members, organizing Zoom interest groups for residents and creating programming, such as exercise sessions broadcast over closed-circuit, in-house television stations. (Graham, 2020, p. 4)

Being present in our sadness is important while at the same time holding as much gratitude or joy as we can. . . . It’s really important for us to be present to the loss as we’re moving through it, but it’s also important to stay present to the restoration, to the moving forward, to the finding the meaning in our living, to allowing moments of joy to come in to release some of the anguish. . . . Crying and screaming are healthy expressions of grief, therapists say, and dancing and singing can also be restorative expressions of emotion. . . . One important element of taking care of yourself is setting boundaries. Being able to say, “today is not the day” when someone comes to you with something you can’t presently deal with . . . noting that women often struggle with feelings that taking care of yourself is somehow selfish. It’s not. (Hunter, 2020, pp. 2-3)

Since action can allay our anxieties, you may want to also consider what you can do to help others who may be more affected by the outbreak than you. Service workers, medical workers, hourly workers and people in the restaurant or entertainment industries may have their livelihoods paralyzed or have to put themselves in disproportionate danger. . . . If you can’t seem to get a handle on your thoughts, professional help can be an option. (Willingham, 2020, pp. 2-3)

“Just having someone to talk to, someone who can help you work through some of these difficult issues, is invaluable. . . . Now more than ever, therapists are becoming indispensable for giving people the tools they need to get through any situation.” (Villano, 2020, p. 4)

Meditation is one tool that can help our immune systems functioning optimally. . . . “Awareness by itself heals. Awareness without conceptual intervention restores self-regulation.” . . . In a 2015 review of studies on the effects of mindfulness-based stress reduction [(MBSR)] and mindfulness-based cognitive therapy (MBCT), researchers found that people who received this therapy were less likely to respond to stressful situations with negative thoughts or unhelpful emotional reactions. Those participants were also more likely to focus on the present moment and less likely to experience ruminating thoughts. Breathing meditations can also reduce muscle tension and your heart rate, which are signs of stress. . . . Breathing meditations are another tool you can add to your coping toolkit, which may also include journaling, baking or virtually connecting with others. (K. Rogers, 2020, pp. 1-3)

The above self-care recommendations are certainly very valuable and have a great deal of potential to benefit many people who are desperately trying to find ways to constructively deal with not only the corona virus pandemic crisis but also the Trump-induced mental health crisis, as described in the preceding section. However, there is one particular self-care recommendation that has not been mentioned by the above authors, and this is one that I personally have gotten much value from and I will discuss in the next section.

## Self-Care and Progressive Politics Through Resisting Trump in the Trump/Coronavirus Era

In this section, I will endeavor to put all the above threads together: self-care, humanistic psychology, progressive politics, the Resisting Trump movement, and the coronavirus, and I will do so through conveying my own continuous involvement in each of these five threads. However, I experience these five threads as interconnecting without any kind of firm boundaries, and therefore my portrayal of my relevant experiences in this regard will encompass all these threads simultaneously.

To begin with, my involvement with progressive politics ever since the November, 2016 U.S. presidential election has been completely immersed with the Resisting Trump movement.^3^ And since 2017, I have been involved in writing articles and giving talks to promote the merging of humanistic psychology with progressive politics via the Resisting Trump movement (Benjamin, 2018a, 2018b, 2019).^1^ However, the recent advent of the horrific coronavirus pandemic crisis has brought things to a whole new level, and I have described my thoughts related to the connections between Trump and the coronavirus (Benjamin, 2020a, 2020b, 2020c) with some preliminary excursions into how these connections can also relate to humanistic psychology (Benjamin, 2020d). For example, I originally had written about the “dangerous leadership and rhetoric” of President Trump (Benjamin, 2019) but this is now extended to the apparent stimulation of hate crimes against Asian Americans related to Trump for a period of time designating the coronavirus as the “Chinese virus” (Benjamin, 2020d; Somvichian-Clausen, 2020).

But the question I want to focus on at this point is how my involvement in progressive politics through the Resisting Trump movement in the current Trump/Coronavirus era goes together with my self-care in the context of humanistic psychology. And much of the answer to this question is related to my involvement with the grass roots progressive politics Resisting Trump Indivisible movement,^3^ which like everything else in these Coronavirus times, has currently transformed into “virtual” connections. I take part in both the relatively small weekly Zoom meetings with my local Bangor, Maine Indivisible group, and in the large monthly National Indivisible conference calls. In regard to my self-care, the weekly Bangor Indivisible meetings consists of only about a dozen people, and I am quite active in voicing my thoughts and feelings, both personal and political. My thoughts and feelings that I have conveyed to my Indivisible colleagues have been largely focused on what it takes to defeat Trump in the November U.S. presidential election, which now means electing Joe Biden to be president (Benjamin, 2020e). For the past year Indivisible was not favorable to Biden, as Biden was not nearly progressive enough to satisfy the progressive leanings of virtually all Indivisible members, which I certainly understand and empathize with.^4^ However, true to their commitment to defeat Trump, Indivisible has now endorsed Biden, with over 95% of Indivisible members voting in favor of the endorsement (Seitz-Wald, 2020).^5^

I have stimulated discussions in my Bangor Indivisible group related to how Indivisible could induce former Bernie Sanders supporters to support Biden, inclusive of my personal disclosure about my communications with my son in this regard (Benjamin, 2020e). I have expressed my concerns, reinforcing the similar concerns expressed by others in the group, about Trump trying to postpone the November presidential election and/or eliminating or minimizing mail-in vote ballots to help him get reelected, through not supporting the continuation of the U.S. post office (Bunch, 2020). I have sparked continued discussions about the sexual assault allegation against Biden, taking into account Biden’s defense of these allegations and the evidence for and against him (Bradner & Lee, 2020; Cillizza, 2020; Dem, 2020). Thus, in the midst of the coronavirus pandemic restrictive environment that we are now required to live through, I have been able to maintain my political/personal connections in a way that has nourished me and made me feel less isolated and more part of the progressive political world. My continued involvement in the Indivisible movement, and in particular with my local Indivisible group virtually, has been a very important part of my self-care as I live through the Trump/Coronavirus era, and is part and parcel of the basic values of humanistic psychology that involve self-expression, authenticity, and empathy.

Furthermore, I have continued my writings regarding the connections of humanistic psychology to progressive politics and the Resisting Trump movement, inclusive of this present article, as I have received constructive feedback and interest from professional humanistic psychology circles in response to my ideas, and what I have to say, about humanistic psychology.^6^ Taken together, the affirming responses that I have received, and am receiving, from Indivisible and professional humanistic psychology circles have the effect of making me feel connected to an idealistic group of people to whom I can relate, which is nourishing my sense of self-care through these turbulent times. This goes hand-in-hand with my personal good fortune to be involved in a continuous loving harmonious (nonsocial distancing) relationship with my wife, as well as with the continuation of my artistic/therapeutic mode of day-to-day functioning, inclusive of my engaging in pure mathematics as a hobby, playing the piano by myself and playing piano/flute with my wife, writing psychology/political articles, enjoying our family dog and cat, engaging in my disciplined mindfulness meditation practice, exercising at home regularly and taking walks, and separating the weekends from the weekdays through continuing to experience homey, fulfilling, and intimate weekends with my wife (Benjamin, 2020d).

This all goes along with stringently practicing social distancing and taking the utmost precautions when I am out in public, while the coronavirus pandemic is still raging, as I recently turned 70 years and I certainly am not taking my good health for granted (Benjamin, 2020d). But all things considered, I am grateful to still be alive and well and have the opportunity to be writing this essay and have it published in a professional humanistic psychology journal. In this way, the five threads of self-care, humanistic psychology, progressive politics, the Resisting Trump movement, and the coronavirus are all woven together for me, with permeable boundaries that are working well to keep me functioning effectively through these trying times. It may be worthwhile for politically like-minded others to also consider finding ways of merging their progressive politics with humanistic psychology in order to enhance their self-care through these turbulent times in the Trump/Coronavirus era. In this regard, I find the following wise and inspiring words from esteemed humanistic psychology elder Maureen O’Hara (2019) to be timely and relevant (Benjamin, 2020d):

I believe that despite the looming threats which often appear to block out the light of hope, we are in fact witnessing a transformative insurgency at the core of which is a new consciousness. There are “persons of tomorrow” everywhere engaged in large and mostly small creative and effective initiatives addressing the multiple challenges humanity faces in the 21st century. . . . It is urgent that as an alternative to the current narratives of despair, fear and division we promote narratives of hope and solidarity not just with other humans but with all the species on the planet. These narratives already exist in the hearts and minds of those who are making a difference. We are in this together. The culture our descendants will inhabit will be created by the actions, stories and choices we make now. (O’Hara, 2019, pp. 147, 149)

## Conclusion

In this essay, I have discussed the relationship of progressive politics to humanistic psychology in the Trump/Coronavirus era. There are harsh realities of personal limitations and challenges to our mental health that we are all facing during these turbulent times. However, there are also a number of self-care practices that we can undertake to help us get through these times, and a number of these practices are consistent with the basic values of humanistic psychology. For me personally, I am continuing to engage in the world of progressive politics and humanistic psychology in the context of the Resisting Trump movement, and along with my good fortune to have a fulfilling personal life, I am consequently undertaking sufficient self-care to be able to function effectively through the crisis that is challenging all of us with our very survival. Along these lines, it may be worthwhile for politically like-minded others to also consider finding ways of merging their progressive politics with humanistic psychology in order to enhance their self-care through these turbulent times in the Trump/Coronavirus era.

## Notes

1. My humanistic psychology Resisting Trump talks, some of which I have presented as a member of the American Psychological Association (APA) Division 32 Humanistic Psychology Task Force on Hate Incidents, have taken place at the APA conventions in 2017 and 2019, the 2019 Association of Humanistic Psychology annual meeting, and at a 2019 London British Association of Humanistic Psychology meeting that I facilitated.

2. See Fox (2020), LaBlanc & Hoffman (2020), Levenson et al. (2020), and Benjamin (2020a, 2020b, 2020c) and the references therein.

3. See Benjamin, E. (2018a, 2018b, 2019) and the references therein.

4. See my essays related to Biden and Trump at www.integralworld.net and www.esperanzacenter.org

5. See the Indivisible detailed contrast between the views of Biden and Trump on a number of issues on the Indivisible homepage at https://2020.indivisible.org/?akid=59083.12450.UrND_Y&rd=1&t=7&utm_medium=link1_20200508&utm_source=email

6. See Benjamin, 2019, 2020d, and Note 1; within the past 2½ years, I have also published an article and reviewed an article for the APA publication *The Humanistic Psychologist* and I have served as a reviewer for two articles in the *Journal of Humanistic Psychology*.
